# Isolated Vastus Lateralis Rupture and Repair Using Suture Anchor Technique

**DOI:** 10.1155/2020/9617303

**Published:** 2020-06-01

**Authors:** Pierce Johnson, Ryan Digiovanni, Tony Nguyen

**Affiliations:** ^1^University of Arizona Phoenix Orthopedics Department, Phoenix, AZ, USA; ^2^The CORE Orthopedics Institute, Phoenix, AZ, USA

## Abstract

**Introduction:**

This is a case report of an isolated vastus lateralis rupture identified by MRI and treated successfully with surgical repair. *Case Presentation*. A 50-year-old male recreational weightlifter who sustained an isolated vastus lateralis rupture while dead lifting and underwent surgical repair using a suture anchor fixation.

**Conclusion:**

An isolated vastus lateralis rupture is a rare injury that may be successfully treated with surgical repair allowing return to preinjury activities.

## 1. Introduction

Anatomically, the vastus lateralis distal fibers combine with the other quadriceps muscles to form the common quadriceps tendon inserting onto the superior pole of the patella. Some of the connective fibers of the vastus lateralis go on to insert onto the lateral side of the patella as well as help form the lateral patellar retinaculum (1). Because of this, the vastus lateralis not only provides additional strength in knee extension force but also provides assistance with patellar stability (2). Quadriceps muscle rupture is a relatively uncommon injury occurring at a reported rate of 1.37/100,000 (3). These injuries are often due to eccentric loading with forced knee extension. They most commonly affect patients over the age of 40 with a higher occurrence in men. There is increased risk of quad tendon rupture with steroid use, fluoroquinolone use, intratendon injections, connective tissue disorders, and systemic disorders such as diabetes, renal disease, and rheumatoid arthritis (3). The most common location of the rupture is at the tendon-patella interface. MRI is the imaging modality of choice to diagnose quadriceps tendon injury due to its high sensitivity and specificity. However, outstanding sensitivities and specificities have been reported for the ultrasound diagnosis of the quadriceps tendon rupture as well (4). There have only been two other reported cases of isolated vastus lateralis tendon rupture (5, 6). This case is the third report of an isolated vastus lateralis rupture.

## 2. Case Presentation

We present a case of a 50-year-old male previously a healthy recreational powerlifter who presented to a clinic several days after sustaining an injury to his left thigh while dead lifting approximately 400 pounds. He was a nonsmoker and otherwise healthy prior to the injury. He described a sudden pop in his left knee with immediate swelling. Examination revealed tenderness to palpation with palpable defect over the lateral quadriceps muscle as well as 3/5 weakness with knee extension and limited knee flexion to approximately 50 degrees. No patellar instability was noted. An MRI was obtained which showed an isolated rupture of the vastus lateralis, and the decision was made to move forward with surgical fixation.

### 2.1. Imaging

**Figure 1:**
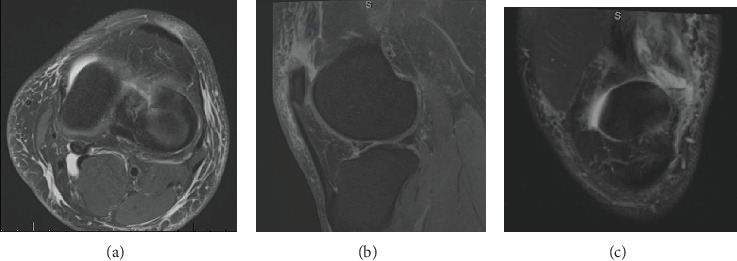
(a) Axial, (b) sagittal, and (c) coronal T2 MRI views showing isolated vastus lateralis tendon rupture with approximately 3 cm proximal retraction.

MRI was performed which showed a full-thickness tear of the vastus lateralis component of the quadriceps tendon from the patellar attachment, retracted by approximately 3 cm. The torn tendon end was diffusely thickened. The vastus medialis, rectus femoris, and vastus intermedius components of the quadriceps tendon were noted to be intact (Figure 1). Operative and nonoperative options along with informed consent were provided. The decision was made to move forward with surgical repair of the tendon as the patient wished to return to preinjury activity level with no weakness.

### 2.2. Surgery

**Figure 2:**
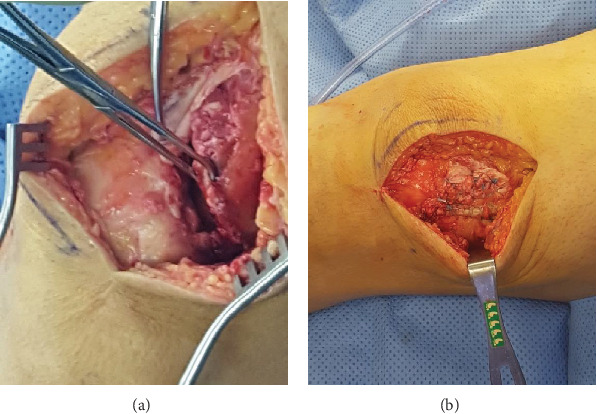
Intraoperative prerepair injury of the isolated vastus lateralis (a). Postrepair intra operative photo of the vastus lateralis (b).

In the operating room, midline incision was placed centered laterally over the palpable defect in the quadriceps tendon. The vastus lateralis was isolated and noted be torn off the lateral third of the patella. This was noted to be a full-thickness tear extending into the knee joint (Figure 2). Of note, the bulk of the quadriceps tendon was still noted to be intact which correlated to the MRI findings. The lateral border of the patella was then prepped for repair. Soft tissue was removed and a bleeding bony bed was prepared. A pilot hole was treated for a 5.5 mm Bio-Corkscrew Suture Anchor (Arthrex Inc., Naples, FL) in the lateral patella. The sutures were then passed through the tendon in the locking Krakow fashion. The knee was placed in hyperextension and the sutures were tied. The split between the quadriceps tendon, and the vastus lateralis were repaired using interrupted figure of eight #2 FiberWire (Arthrex Inc., Naples, FL). The lateral retinaculum was noted to be torn and this was repaired using interrupted #2 FiberWire (Arthrex, Naples, FL). The knee was flexed and was stable to about 80 degrees. Fascia was closed with 0 Vicryl. The skin was closed with 2-0 Monocryl and a running 3-0 Monocryl.

### 2.3. Follow-up

Postoperatively, the patient was made to perform full weight bearing with hinged knee brace initially locked in extension during ambulation. He was gradually advanced to an unrestricted range of motion at 6 weeks with continued physical therapy for strength and range of motion exercises. At approximately 10 weeks after his injury, the patient exhibited 5/5 strength in knee extension and near normal knee range of motion. No palpable gap was detected. He was allowed to gradually return to full activity starting at 3 months with full activity achieved without restrictions at 4 months postinjury.

## 3. Discussion

Quadriceps tendon rupture is a relatively uncommon injury. Isolated vastus lateralis ruptures are exceedingly rare with only 2 reported cases (5, 6). In both of these reported cases, the patients underwent successful repair using a suture anchor technique. This was similar in this patient. Another option for tendon repair is the creation of patellar bone tunnels and the use of transosseous sutures. The downside of this technique is that it requires more soft tissue dissection, larger incision, and potentially longer recovery times. Lighthart et al. performed a biomechanical study evaluating repair strength suture anchors and transosseous sutures for quadriceps rupture repair and found no difference in repair strength after 1000 cycles (7).

We believe the case presented here not only adds to the existing literature but also highlights an effective method of surgical repair using the suture anchor technique allowing the patient to return to the preinjury level of activity.

## 4. Conclusion

Isolated vastus lateralis rupture is a rare injury that may be successfully treated with suture anchor repair allowing the return to preinjury activities. This case report brings awareness to this type of injury and adds to the limited existing literature. Although isolated injuries such as the one presented are extremely rare and pose a diagnostic challenge, it is important to keep in mind in order to provide the best care for the patient.
